# Contributions to Obstetric Jurisprudence

**Published:** 1859-09

**Authors:** Horatio R. Storer

**Affiliations:** Boston


ART. I.—*Contributions to Obstetric Jurisprudence.* By HORATIO R. STORER, M.D., of Boston.

### CRIMINAL ABORTION. (CONTINUED.)

### VII. ITS OBSTACLES TO CONVICTION.

WE have already seen that there are special, though, it is to be hoped, not wholly insurmountable causes for the existing prevalence of abortion. It now becomes our duty to consider some of these reasons in detail, in so far as they relate to and obstruct the course of justice.

It would seem, from what has been previously said, that little doubt could be entertained of the inefficacy of our present statutes against abortion. There are few of the States whose laws on this point are so wisely and completely drawn as in Massachusetts; yet, as they there stand, they cannot, as such, be enforced. In that Commonwealth, according to the reports of the attorney-general, during the eight years from 1849 to 1857, omitting 1853—as there seems to have been no report rendered for that year—there were, as we have seen, 32 trials for abortion, and not a single conviction!

A committee of the State Medical Society of Massachusetts, to whom the propriety of a professional appeal to the Legislature for more protective statutes had been referred by the District Society of Boston, having reported against such action, on the ground "that the laws of the Commonwealth are already sufficiently stringent, provided that they are executed,"* it becomes the more necessary for us to strike at the root of the whole matter, and to show, if possible, why conviction, unless in case of the death of the mother, cannot at present be obtained.

It has been thought, even publicly argued, that in the fact that statutes against abortion are almost everywhere not only not enforced, but not attempted to be enforced, there is afforded strong evidence of the existence of an ultimate and absolute impossibility of thus meeting the crime. The idea, though a fallacious one, is yet attributable to an important and evident cause.

That the prevalence of abortion is in great measure owing to ignorance of guilt, on the part of the community at large, we have shown. We now assert that its futile prohibition by the law, its toleration, are plainly in consequence of similar ignorance on the part of legislators, and of officers of justice.

Our communities form their own laws, and, therefore, as was pointed out at the commencement of our remarks, these must necessarily bear the stamp of public opinion; while the officers by whom they are to be enforced—juror, attorney, judge—looking to the only source possible for their enlightenment on this subject, to medical men, have hitherto found but few bold and honest statements,† and these unindorsed by the mass of the profession; or, in the total silence, a practical sanction of the popular belief. This is no exaggeration; the assertion is fully borne out by facts. Need we wonder, then, that the laws are not enforced, that indeed their enforcement is not attempted? But this first and great cause, it is apparent, is by no means an essential one.

We need add nothing to what we have already said, of those obstacles to conviction, arising from circumstances common in greater or less degree to other crimes;—the difficulties of detection and of obtaining proof,

---

* Medical Communications of the Mass. Med. Soc., 1858, p. 77.

The writer having been a member of this committee, here enters, as he has already done by letter to the councillors of the Society, his earnest protest against the plainly erroneous opinion avowed in that report, which was presented and accepted during his absence from the State.

By the laws of Massachusetts, the offence is considered as mainly against the person of the mother. In case of her death, already sufficiently provided for at common law, convictions can be effected, with great difficulty, under the statute,—as has twice occurred the present year, in the cases of Jackson and Brown; but hardly otherwise.

† In this connection honorable mention is due Drs. TATUM and JOYNES, of Virginia, for their papers on "The Attributes of the Impregnated Germ," and "Some of the Legal Relations of the Fœtus in Utero" (Virginia Medical Journal, 1856.) Through the agency of the latter of these gentlemen, an important modification has been made in the law of the State; as has also been effected in Wisconsin, by Dr. BRISBANE.

however great these are allowed to be,—but we proceed at once to consider the laws themselves by which in this country the crime of abortion is attempted, or is expected to be suppressed.*

The arguments by which we have shown the mistaken premise on which the common law of England, as covering this crime, is founded, and by adoption, the common law of this country, will not have been forgotten. We shall perceive that similar reasoning applies with equal force to the special statutes, where such exist, of almost every State in the Union.

In the following States, Rhode Island, New Jersey, Pennsylvania, Delaware, Maryland, North Carolina, South Carolina, Georgia, Florida, Kentucky, Tennessee, Iowa, and the District of Columbia, there appear to exist no statutes against abortion, and the crime can only be reached at common law and by the rulings of the courts.

In the case of the District of Columbia, all rulings are based on the common law, the old English statutes, from Elizabeth to George II., and the old colonial statutes of the Province of Maryland, down to 1800, the period of cession to the United States of that portion of Maryland lying on the north side of the Potomac, now included in the federal district.

The later English statutes, even those of George IV., being enacted subsequent to the separation from the mother country, are not recognized in the District. In the State courts, so far as the rules and principles of the common law are applicable to the administration of criminal law, and have not been altered or modified by acts of the colonial or provincial government, or by the State Legislature, they have the same force and effect as laws formally enacted.† In the States referred to, therefore, as having no special statutes of their own, the later English statutes, though not of absolute force, are to a certain extent undoubtedly acknowledged.

By the common law of England, as stated by Blackstone :—

"If a woman is quick with child, and by a potion or otherwise, killeth

* For valuable information in this connection, I am indebted to many friends, more particularly to Drs. THAYER, of New Hampshire, PHELPS, of Vermont, CHAS. HOOKER, of Connecticut, BLATCHFORD, of New York, WOOD, of Pennsylvania, THOMPSON, of Delaware, WROTH, of Maryland, BRAINARD, of Illinois, CAMERON, of Indiana, LELAND, of Michigan, LE BOUTILLIER, of Minnesota, BRISBANE, of Wisconsin, POPE, of Missouri, HOYT, of Tennessee, HAXALL and JOYNES, of Virginia, SEMMES, of District of Columbia, DICKSON, of North Carolina, LOPEZ, of Alabama, BARTON, of Louisiana, (now of South Carolina,) and to my relatives, WOODBURY and BELLAMY STORER, Esqrs., of Maine and Ohio, and JAMES M. KEITH, Esq., of Boston, late District-Attorney for Norfolk and Plymouth Counties. In every instance, however, verification of the statutes has been made from copies in the State Library of Massachusetts.

† DAVIS, Criminal Justice, p. 482.

it in her womb, or if any one beat her, whereby the child dieth in her body, and she is delivered of a dead child, this, though not murder, was by the ancient law homicide, or manslaughter. But the modern law doth not look upon this offence in quite so atrocious a light, but merely as a heinous misdemeanor."* "But if the child be born alive, and afterwards die in consequence of the potion or beating, it will be murder."†

By this law it is necessary to furnish proof not merely of pregnancy, but of quickening; no one besides the mother can be reached, save in the rare instance of beating; it is often impossible to prove that a child, born living, has died in consequence of means used upon itself or its mother before the birth.

The Ellenborough act,‡ passed in 1803, runs thus:—

"If any person shall willfully and maliciously administer to, or cause to be administered to, or take any medicine, drug, or other substance or thing whatsoever, or use, or cause to be used or employed, any instrument, etc., with intent to procure the miscarriage of any woman, not being, or not being proved to be, quick with child at the time of committing such thing, or using such means, then, and in every such case, the persons so offending, their counselors, aiders and abettors, shall be and are declared guilty of felony, and shall be liable," etc. etc.; if before quickening, to fine, the pillory, stripes, or transportation; if after quickening, to death;—but in the clause providing for the latter case, mention of instruments was omitted.

Punishment was thus extended to the crime prior to quickening; and besides the actual perpetrator, to those counseling and assisting therein. For its commission after quickening, capital punishment was restored; but by a strange oversight, no penalty was provided for the cases occurring at this period, where the abortion was induced by instrumental or other mechanical violence.

To remedy this defect another act, that of Lord Lansdowne,§ was substituted in 1828:—

"If any person, with intent to procure the miscarriage of any woman then being quick with child, unlawfully and maliciously shall administer to her, or cause to be taken by her, any poison or other noxious thing, or shall use any instrument, or other means whatever, with the like intent; every such offender, and every person counseling, aiding or abetting such offender, shall be guilty of felony, and being convicted thereof, shall suffer death as a felon;" if the woman were "not, or not proved to be, then

* 1 Commentaries, 129.

† Ibid.

‡ 43 George III., c. 58.

§ 9 Geo. IV., c. 31; 10 Geo. IV., c. 34.

quick with child," the offence was still felony, and punished by transportation, or imprisonment and stripes.

By this enactment, the mistaken belief in a difference of guilt, according to the period of fœtal life, was still retained. For which reason, and in the false hope that by again abolishing capital punishment, juries would more frequently decide in accordance with fact, the English law has later, and during the present reign, still further been modified and rendered more just, simple, and comprehensive.*

"Whosoever, with the intent to procure the miscarriage of any woman, shall unlawfully administer to her, or cause to be taken by her, any poison or other noxious thing, or shall unlawfully use any instrument, or other means whatsoever, with the like intent, shall be guilty of felony, and being convicted thereof, shall be liable," etc. etc., to transportation or imprisonment.

By the present English statute, the woman herself can hardly yet be reached; many convictions must still be lost from failure to prove the agent either a poison or noxious thing; in other respects, however, it is well drawn. Attempts at the crime are covered, and proof is not required of pregnancy, or of actual injury to the mother.

So far the instances in this country of an absence of special statutes. Where such exist, they may be variously classified. Reserving for a little all other considerations, we find them at once falling into four great divisions.

I. Those acknowledging the crime only after quickening has occurred:—

| | |
|---|---|
| Connecticut, | Minnesota, |
| Mississippi, | Oregon. |
| Arkansas, | |

II. Those acknowledging the crime throughout pregnancy, but supposing its guilt to vary with the period to which this has advanced:—

| | |
|---|---|
| Maine, | Ohio, |
| New Hampshire, | Michigan.† |
| New York, | |

III. Those acknowledging the crime throughout pregnancy, unmitigated; but still requiring proof of the existence of this state:—

| | |
|---|---|
| Vermont, | Missouri, |
| Massachusetts, | Alabama, |

* 7 William IV.; 1 Vict., c. 85.

† To this list may also be added the Territory of Washington.

| | |
|---|---|
| Illinois, | Louisiana, |
| Wisconsin, | Texas, |
| Virginia, | California.* |

IV. That, like the present English statute, requiring no such proof, and punishing also the attempt, even though pregnancy do not exist :—

Indiana.

Briefly to recapitulate these groups :—

| | | | |
|---|---|---|---|
| Maine, | Class II. | Alabama, | Class III. |
| New Hampshire, | " II. | Mississippi, | " I. |
| Vermont, | " III. | Louisiana, | " III. |
| Massachusetts, | " III. | Texas, | " III. |
| Rhode Island, | no statute. | Ohio, | Class II. |
| Connecticut, | Class I. | Indiana, | " IV. |
| New York, | " II. | Illinois, | " III. |
| New Jersey, | none. | Michigan, | " II. |
| Pennsylvania, | none. | Kentucky, | none. |
| Delaware, | none. | Tennessee, | none. |
| Maryland, | none. | Missouri, | Class III. |
| District of Columbia, | none. | Arkansas, | " I. |
| Virginia, | Class III. | Wisconsin, | Class III. |
| North Carolina, | none. | Iowa, | none. |
| South Carolina, | none. | Minnesota, | Class I. |
| Georgia, | none. | California, | " III. |
| Florida, | none. | Oregon, | " I. |

We now proceed to the consideration of these statutes in detail, and for this purpose, present them at length.

I. States acknowledging the crime only after quickening :—

CONNECTICUT.—"Every person who shall willfully and maliciously administer to, or cause to be administered to, or taken by, any woman then being quick with child, any medicine, drug, noxious substance, or other thing, with intent thereby to produce the miscarriage of such woman, or to destroy the child of which she is pregnant, or shall willfully and maliciously use and employ any instrument or other means to produce such miscarriage, or to destroy such child, shall suffer imprisonment in the Connecticut State prison for a term not less than seven, nor more than ten years."†

MISSISSIPPI.—"The willful killing of an unborn quick child, by any injury to the mother of such child, which would be murder if it resulted in the death of the mother, shall be deemed manslaughter.

* The Territory of Kansas belongs to the above group.

† Compiled Statutes of Connecticut, 1854, p. 307.

"Every person who shall administer to any woman pregnant with a quick child, any medicine, drug, or substance whatever, or shall use or employ any instrument or other means, with intent thereby to destroy such child, unless the same shall have been necessary to preserve the life of such mother, or shall have been advised by a physician to be necessary for such purpose, shall be deemed guilty of manslaughter."* Punishment, by fine not less than one thousand dollars, or imprisonment in the county jail for not more than one year, or in the penitentiary for not less than two years.

ARKANSAS.—"The willful killing of an unborn quick child, by any injury to the mother of such child, which would be murder if it resulted in the death of such mother, shall be adjudged manslaughter.

"Every person who shall administer to any woman pregnant with a quick child, any medicine, drug, or substance whatever, or shall employ any instrument or other means, with intent thereby to destroy such child, and thereby shall cause its death, unless the same shall be necessary to preserve the life of the mother, or shall have been advised by a regular physician to be necessary for such purpose, shall be deemed guilty of manslaughter."†

MINNESOTA.—"The willful killing of an unborn infant child, by any injury to the mother of such child, which would be murder if it resulted in the death of such mother, shall be deemed manslaughter in the first degree.

"Every person who shall administer to any woman pregnant with a quick child, any medicine, drug, or substance whatever, or shall use or employ any instrument or other means, with intent thereby to destroy such child, unless the same shall have been necessary to preserve the life of such mother, or shall have been advised by two physicians to be necessary for such purpose, shall, in case the death of such child or of such mother be thereby produced, be deemed guilty of manslaughter in the second degree."‡ Punishment for first degree, imprisonment in the territorial prison for not less than seven years; and for second degree, not more than seven years nor less than four.

OREGON.—"Any person who shall administer to any woman pregnant with a quick child, or prescribe for any such woman, or advise or procure any such woman to take any medicine, drug, or substance whatever, or shall use or employ any instrument or other means, with intent thereby to destroy such child, unless the same shall have been necessary to pre-

* Revised Code of Mississippi, 1857, chap. 64, p. 601.

† Digest of Statutes of Arkansas, 1848, chap. 51, p. 325.

‡ Revised Statutes of Minnesota, 1851, chap. 100, p. 493.

serve the life of such mother, shall, in case the death of such child, or of such mother, be thereby produced, be deemed guilty of manslaughter."* Punishment, imprisonment in the penitentiary for not less than one or more than ten years, and by fine not exceeding five thousand dollars.

II. Those acknowledging the crime throughout pregnancy, but as of different degrees of guilt:—

MAINE.—"Whoever administers to any woman pregnant with child, whether such child is quick or not, any medicine, drug, or other substance, or uses any instrument or other means, unless the same were done as necessary for the preservation of the mother's life, shall be punished, if done with intent to destroy such child, and thereby it was destroyed before birth, by imprisonment not more than five years, or by fine not exceeding one thousand dollars; if done with intent to procure the miscarriage of such woman, by imprisonment less (*sic*) than one year, and by fine not exceeding one thousand dollars."†

NEW HAMPSHIRE.—"Every person who shall willfully administer to any pregnant woman, any medicine, drug, substance, or thing whatever, or shall use or employ any instrument or means whatever, with intent thereby to procure the miscarriage of any such woman, unless the same shall have been necessary to preserve the life of such woman, or shall have been advised by two physicians to be necessary for that purpose, shall, upon conviction, be punished by imprisonment in the county jail not more than one year, or by a fine not exceeding one thousand dollars, or by both such fine and imprisonment, at the discretion of the court.

"Every person who shall administer to any woman pregnant with a quick child, any medicine, drug, or substance whatever, or shall use or employ any instrument or means whatever, with intent thereby to destroy such child, unless the same shall have been necessary to preserve the life of such woman, or shall have been advised by two physicians to be necessary for such purpose, shall, upon conviction, be punished by fine not exceeding one thousand dollars, and by confinement to hard labor not less than one year nor more than ten years.

"Any person who shall cause the death of any pregnant woman, in the perpetration or attempt to perpetrate either of the crimes mentioned in the two preceding sections, or in consequence of the perpetration or the attempt to perpetrate either of said crimes, shall be taken and deemed to be guilty of murder in the second degree, and be punished accordingly.

"Any woman who shall voluntarily submit to the violation of the provisions of this act (this and the three preceding sections‡) upon herself,

---

* Statutes of Oregon, 1855, chap. 3, p. 310.

† Revised Statutes of Maine, 1857, chap. 124, p. 685.

‡ The above should evidently read "the first two sections," to be possible.

shall be punished by imprisonment in the county jail not exceeding one year, or by fine not exceeding one thousand dollars, or by both said fine and imprisonment, at the discretion of the court."*

NEW YORK.—"The willful killing of an unborn quick child, by an injury to the mother of such child, which would be murder if it resulted in the death of such mother, shall be deemed manslaughter in the first degree.

"Every person who shall administer to any woman pregnant with a quick child, or prescribe for any such woman, or advise or procure any such woman to take any medicine, drug, or substance whatever, or shall use or employ any instrument or other means, with intent thereby to destroy such child, unless the same shall have been necessary to preserve the life of such mother, shall, in case the death of such child, or of such mother be thereby produced, be deemed guilty of manslaughter in the second degree.

"Every person who shall administer to any pregnant woman, or prescribe for any such woman, or advise or procure any such woman to take any medicine, drug, substance, or thing whatever, or shall use or employ any instrument, or other means whatever, with intent thereby to procure the miscarriage of any such woman, shall, upon conviction, be punished by imprisonment in a county jail not less than three months nor more than one year.

"Every woman who shall solicit of any person any medicine, drug, or substance, or thing whatever, and shall take the same, or shall submit to any operation, or other means whatever, with intent thereby to procure a miscarriage, shall be deemed guilty of a misdemeanor, and shall, upon conviction, be punished by imprisonment in the county jail not less than three months nor more than one year, or by a fine not exceeding one thousand dollars, or by both such fine and imprisonment."†

OHIO.—"Any physician, or other person, who shall willfully administer to any pregnant woman, any medicine, drug, substance, or thing whatever, or shall use any instrument, or other means whatever, with intent thereby to procure the miscarriage of any such woman, unless the same shall have been necessary to preserve the life of such woman, or shall have been advised by two physicians to be necessary for that purpose, shall, upon conviction, be punished by imprisonment in the county jail not more than one year, or by fine not exceeding five hundred dollars, or by both such fine and imprisonment.

"Any physician, or other person, who shall administer to any woman,

---

* Compiled Statutes of New Hampshire, 1853, chap. 227, p. 544.

† Revised Statutes of New York, 1852, ii. pp. 847, 876. The last section of this statute does not require proof of pregnancy.

pregnant with a quick child, any medicine, drug, or substance whatever, or shall use or employ any instrument, or other means, with intent thereby to destroy such child, unless the same shall have been necessary to preserve the life of such mother, or shall have been advised by two physicians to be necessary for such purpose, shall, in case of the death of such child or mother, in consequence thereof, be deemed guilty of a high misdemeanor, and upon conviction thereof, shall be imprisoned in the penitentiary not more than seven years, nor less than one year."*

MICHIGAN.—"The willful killing of an unborn quick child, by any injury to the mother of such child, which would be murder if it resulted in the death of such mother, shall be deemed manslaughter.

"Every person who shall administer to any woman pregnant with a quick child, any medicine, drug, or substance whatever, or shall use or employ any instrument, or other means, with intent thereby to destroy such child, unless the same shall have been necessary to preserve the life of such mother, or shall have been advised by two physicians to be necessary for such purpose, shall, in case the death of such child or of such mother be thereby produced, be deemed guilty of manslaughter.

"Every person who shall willfully administer to any pregnant woman, any medicine, drug, substance, or thing whatever, or shall employ any instrument, or other means whatever, with intent thereby to procure the miscarriage of any such woman, unless the same shall have been necessary to preserve the life of such woman, or shall have been advised by two physicians to be necessary for that purpose, shall, upon conviction, be punished by imprisonment in a county jail not more than one year, or by a fine not exceeding five hundred dollars, or by both such fine and imprisonment."†

---

* Revised Statutes of Ohio, 1854, chap. 162, p. 296.

† Compiled Laws of Michigan, 1857, vol. ii. chap. 180, p. 1509. The statute of the Territory of WASHINGTON is very similar to those above.

"Every person who shall administer to any woman pregnant with a quick child, any medicine, drug, or substance whatever, or shall use or employ any instrument, or other means, with intent thereby to destroy such child, unless the same shall have been necessary to preserve the life of such mother, shall, in case the death of such child or of such mother be thereby produced, on conviction thereof, be imprisoned in the penitentiary not more than twenty years, nor less than one year.

"Every person who shall administer to any pregnant woman, or to any woman whom he supposes to be pregnant, any medicine, drug, or substance whatever, or shall use or employ any instrument, or other means, thereby to procure the miscarriage of such woman, unless the same is necessary to preserve her life, shall, on conviction thereof, be imprisoned in the penitentiary not more than five years, nor less than one year, or be imprisoned in the county jail not more than twelve months, nor less than one month, and be fined in any sum not exceeding one thousand dollars." Statutes of the Territory of Washington, 1855, p. 81.

III. Those not allowing this mitigation, but still requiring proof of pregnancy:—

VERMONT.—"Whoever maliciously, or without lawful justification, with intent to cause and procure the miscarriage of a woman, then pregnant with child, shall administer to her, prescribe for her, or advise or direct her to take or swallow any poison, drug, medicine, or noxious thing, or shall cause, or procure her, with like intent, to take or swallow any poison, drug, medicine, or noxious thing; and whoever maliciously and without lawful justification, shall use any instrument, or means whatever, with the like intent, and every person, with the like intent, knowingly aiding and assisting such offenders, shall be deemed guilty of felony, if the woman die in consequence thereof, and shall be imprisoned in the State prison not more than ten years, nor less than five years; and if the woman does not die in consequence thereof, such offenders shall be deemed guilty of a misdemeanor, and shall be punished by imprisonment in the State prison not exceeding three years, nor less than one year, and pay a fine not exceeding two hundred dollars."*

MASSACHUSETTS.—"Whoever maliciously or without lawful justification, with intent to cause and procure the miscarriage of a woman then pregnant with child, shall administer to her, prescribe for her, or advise or direct her to take or swallow any poison, drug, medicine, or noxious thing, or shall cause or procure her, with like intent, to take or swallow any poison, drug, medicine, or noxious thing; and whoever maliciously and without lawful justification shall use any instrument, or means whatever, with the like intent, and every person with the like intent knowingly aiding and assisting such offender or offenders, shall be deemed guilty of felony, if the woman die in consequence thereof, and shall be imprisoned not more than twenty years, nor less than five years, in the State prison; and if the woman doth not die in consequence thereof, such offender shall be guilty of a misdemeanor, and shall be punished by imprisonment not exceeding seven years, nor less than one year, in the State prison, or house of correction, or common jail, and by fine not exceeding two thousand dollars."†

ILLINOIS.—"Every person who shall administer, or cause to be administered or taken, any noxious or destructive poison, substance, or liquid, with the intention to procure the miscarriage of any woman then being with child, and shall thereof be duly convicted, shall be imprisoned for a term not exceeding three years in the penitentiary, and fined in a sum not exceeding one thousand dollars."‡

* Compiled Statutes of Vermont, 1850, chap. 108, p. 560.

† Supplement to the Revised Statutes of Massachusetts, 1849, p. 322.

‡ Statutes of Illinois, 1858, vol. i. p. 381.

WISCONSIN.—"Every person who shall administer to any pregnant woman, or prescribe for any such woman, or advise or procure any such woman to take any medicine, drug, or substance, or thing whatever, or shall use or employ any instrument, or other means whatever, or advise or procure the same to be used, with intent thereby to procure the miscarriage of any such woman, shall, upon conviction, be punished by imprisonment in a county jail, not more than one year nor less than three months, or by fine not exceeding five hundred dollars, or by both fine and imprisonment, at the discretion of the court.

"Every woman who shall take any medicine, drug, substance, or thing whatever, or who shall use or employ any instrument, or shall submit to any operation, or other means whatever, with intent to procure a miscarriage, shall, upon conviction, be punished by imprisonment in a county jail not more than six months, nor less than one month, or by a fine not exceeding three hundred dollars, or by both fine and imprisonment, at the discretion of the court."*

VIRGINIA.—"Any free person who shall administer to, or cause to be taken by a woman, any drug or other thing, or use any means, with intent to destroy her unborn child, or to produce abortion or miscarriage, and shall thereby destroy such child, or produce such abortion or miscarriage, shall be confined in the penitentiary not less than one, nor more than five years. No person, by reason of any act mentioned in this section, shall be punishable where such act is done in good faith, with the intention of saving the life of such woman or child."†

MISSOURI.—"Every physician, or other person, who shall willfully administer to any pregnant woman, any medicine, drug, or substance whatsoever, or shall use or employ any means whatsoever, with intent thereby to procure abortion, or the miscarriage of any such woman, unless the same shall have been necessary to preserve the life of such woman, or shall have been advised by a physician to be necessary for that purpose, shall, upon conviction, be adjudged guilty of a misdemeanor, and punished by imprisonment in the county jail not exceeding one year, or by fine not exceeding five hundred dollars, or by both such fine and imprisonment."‡

ALABAMA.—"Any person who willfully administers to any pregnant woman, any drug or substance, or uses and employs any instrument or other means to procure her miscarriage, unless the same is necessary to preserve her life, and done for that purpose, must, on conviction, be fined

---

* Revised Statutes of Wisconsin, 1858, chap. 169, sect. 58. It will be noticed that the second section of the above statute differs from the first, in not requiring the proof of pregnancy.

† Code of Virginia, 1849, chap. 191, p. 724.

‡ Revised Statutes of Missouri, 1856, i. chap. 50, p. 567.

not more than five hundred dollars, and imprisoned not less than three or more than twelve months."*

LOUISIANA.—"Whoever shall feloniously administer, or cause to be administered, any drug, potion, or any other thing, to any woman, for the purpose of procuring a premature delivery, and whoever shall administer, or cause to be administered, to any woman pregnant with child, any drug, potion, or any other thing, for the purpose of procuring abortion, or a premature delivery, shall be imprisoned at hard labor for not less than one, nor more than ten years."†

TEXAS.—"If any person shall designedly administer to a pregnant woman, with her consent, any drug or medicine, or shall use toward her any violence, or any means whatever, externally or internally applied, and shall thereby procure an abortion, he shall be punished by confinement in the penitentiary not less than two, nor more than five years; if it be done without her consent the punishment shall be doubled.

"Any person who furnishes the means for procuring an abortion, knowing the purpose intended, is guilty as an accomplice.

"If the means used shall fail to produce an abortion, the offender is nevertheless guilty of an attempt to procure abortion, provided it be shown that such means were calculated to produce that result, and shall receive one half the punishment prescribed.

"If the death of the mother is occasioned by an abortion so produced, or by an attempt to effect the same, it is murder.

"If any person shall, during parturition of the mother, destroy the vitality or life in a child, which child would otherwise have been born alive, he shall be punished by confinement in the penitentiary for life, or any period not less than five years, at the discretion of the jury.‡

"Nothing contained in this chapter shall be deemed to apply to the case of an abortion procured or attempted to be procured by medical advice for the purpose of saving the life of the mother."§

CALIFORNIA.—"Every person who shall administer or cause to be administered or taken, any medicinal substance, or shall use, or cause to be used, any instrument whatever, with the intention to procure the miscar-

---

* Code of Alabama, 1852, sect. 3230, p. 582.

† Revised Statutes of Louisiana, 1856, p. 138. By its wording, this statute might be forced into the next division.

‡ I insert this clause not merely for its relation to the points we are now considering, but for its important bearing on the broad question of infanticide during labor; concerning which it stands in bold and direct antagonism to all the rulings of the common law in this country and abroad. In other respects also, though not faultless, the Texas statute is rationally and admirably drawn.

§ Penal Code of Texas, 1857, p. 103.

riage of any woman then being with child, and shall be thereof duly convicted, shall be punished by imprisonment in the State prison for a term not less than two years, nor more than five years; provided, that no physician shall be affected by this section, who, in the discharge of his professional duties, deems it necessary to produce the miscarriage of any woman in order to save her life."*

IV. The State, like England, where proof of pregnancy is not required:†

INDIANA.—"Every person who shall willfully administer to any pregnant woman, or to any woman whom he supposes to be pregnant, anything whatever, or shall employ any means with intent thereby to procure the miscarriage of such woman, unless the same is necessary to preserve her life, shall be punished by imprisonment in the county jail not exceeding twelve months, and be fined not exceeding five hundred dollars."‡

The impossibility of proving the existence of pregnancy, in all cases of early occurring, and in many of advanced abortion, save by its result, is undeniable. Quickening, where abortion is criminal, is seldom previously ascertained by witnesses, at least from actual examination. If the mother is dead, her own testimony, not often willingly given, is lost, and the only reliable evidence that the usual period of quickening had been reached, is from the body of the fœtus, frequently concealed or destroyed. The law requires, in proof of the existence of quickening, that the woman should herself have felt the child move within her;§

* Digest of Laws of California, 1857, art. 1905, p. 334. The statute of the Territory of KANSAS, similar to the above, is as follows:—

"Every physician or other person who shall willfully administer to any pregnant woman, any medicine, drug, or substance whatever, or shall use or employ any instrument or means whatsoever, with intent thereby to procure abortion, or the miscarriage of any such woman, unless the same shall have been necessary to preserve the life of such woman, or shall have been advised by a physician to be necessary for that purpose, shall, upon conviction, be adjudged guilty of a misdemeanor, and punished by imprisonment in a county jail not exceeding one year, or by fine not exceeding five hundred dollars, or by both such fine and imprisonment." Statutes of Kansas, 1855, chap. 48, p. 243.

† We have already commented upon the phraseology of the Louisiana statute. The latitude of its first clause is shown by the context to have been unintentional, and therefore hardly justifies a change in its classification. The second section of the Statute of Washington Territory, however, is closely analogous to that now given; while the final sections of the statutes both of New York and Wisconsin, which make it penal for a woman voluntarily to effect or submit to the unjustifiable induction of abortion, are equally silent regarding proof of the existence of pregnancy.

‡ Revised Statutes of Indiana, 1852, p. 437.

§ Rex *vs.* Phillips, 3 CAMPBELL, 77; RUSSELL, Crim. Law, 553–4; 1 GABBETT, Crim. Law, 522; 1 BISHOP, Crim. Law, 386.

thus discarding* the distinction once very properly made by a learned judge, "Quick with child, is having conceived; with quick child, is where the child is quickened."†

We have already sufficiently insisted on the error, injustice, and actual wrong to society, of making this absurd distinction between the fœtus of an early and a later age, and only refer to it at the present time as foremost among the existing obstacles to conviction.

In some of the States, the offence is considered a trifling one, except as affecting the person or life of the mother; this is the case in New Hampshire, Vermont, and Massachusetts. In Ohio, Michigan, Minnesota, Wisconsin, and Oregon, the proved death of either child or mother is required to make abortion a high misdemeanor, felony or manslaughter; while in Virginia and Arkansas it is necessary to constitute an indictable offence, in the one State that the death of the child should be proved, and in the other that this or its premature discharge has actually taken place. An attempt at the crime would here seem beyond indictment, unless fœtal life were destroyed; though if this could be proved, the attempt might perhaps be reached, even though the fœtus were retained in utero, and a true abortion, its discharge, had not taken place. In Maine, the fœtus must have died before birth; if, born living, it yet die in consequence of the abortion, the crime would seem not indictable, save at common law. In other States, allowing the fact of pregnancy to be proved, and in Indiana alone this is not necessary, attempts at abortion are as indictable as the act consummated, save in Texas, where the means used must be shown to be such as "were calculated to produce the result."

In but few instances is the crime, intrinsically considered, accounted a heinous one, and recognized in its true character—an attempt to destroy the life of the child. In Texas, the consent of the mother half palliates the crime; while in very many codes abortion is omitted from the list of offences against the person, and accounted only a breach of public decency and morality.

In Ohio, as we have seen, it is called a high misdemeanor; in New York, Michigan, Oregon, Arkansas, and Mississippi, it is styled manslaughter in the first degree. The punishment inflicted by the latter of these States, however, is ridiculously trivial, and in all of them proof of quickening is required. In New York it has been determined that under an indictment for procuring the abortion of a quick child, which by the revised statutes is a felony, the prisoner may be convicted, though it turn out that the child was not quick, and the offence therefore a mere misde-

* State *vs.* Cooper, 2 ZABRISKIE, 52, 57; Rex *vs.* Russell, 1 MOODY, 356, 360.

† Regina *vs.* Wycherley, 8 CARRINGTON and PAYNE, 265.

meanor;* as in the remaining States it is indeed either in name or by penalty considered,—a simple, trivial, and venial offence.

But it must not be forgotten that the true nature of manslaughter consists in the absence of all malice or willful intent, expressed or implied, to do personal injury: out of tenderness to the frailty of human nature,† the law mercifully denying to such homicide, from unlawful accident or hasty passion, the same degree of guilt with the cool, deliberate act;‡ nor that misdemeanors are specifically confined to the following category: disturbances of the public peace, trivial personal injuries, public nuisances and scandals, lewdness, and incentives to special crimes.§ It is evident that under neither of these heads can the crime of abortion be properly made to fall.

Difficulties of conviction, similar to those we have seen obtaining in England, and arising from requirement of proof that the means employed are unlawful, also present themselves in several of our States, though in most they have been wisely avoided. In Vermont and Massachusetts the agent administered, to be indictable, must have been a "poison, drug, medicine, or noxious thing;" in Illinois, a "poison, noxious, or destructive substance, or liquid;" in Texas, a "drug or medicine;" and in California, a "medicinal substance." In Maine, New Hampshire, Connecticut, New York, Ohio, Michigan, Minnesota, Wisconsin, Oregon, Virginia, Missouri, Arkansas, Alabama, Mississippi, and Louisiana, the wording of the statute is sufficiently comprehensive; while in Indiana, there is used the curt but significant expression "anything whatever," it not being necessary under this statute, in an indictment for administering medicine or any other substance, to procure abortion, for the agent to be described as noxious, or even its name to be stated.|| The use of instrumental and other violence is generally well provided against.

The truth is, as has indeed been ruled, that it should not be necessary to show, in pleading or evidence, that the drug, etc. administered is noxious or the like, the intent to procure abortion being the gist of the attempt;¶ and if a person administer a bit of bread merely, with this intent, it is sufficient to constitute the offence.** On the other hand, it has been laid down, that if the thing administered could of itself by no possi-

* The People *vs.* Jackson, 3 HILL, N. Y. Reports, 92; WHARTON, Criminal Law, 98.

† DAVIS, Crim. Justice, 484.

‡ WHARTON, Amer. Crim. Law, 424.

§ WHARTON, Amer. Crim. Law, 75.

|| The State *vs.* Vawter, 7 BLACKFORD, 592.

¶ 1 GABBETT, Crim. Law, 523; ARCHBOLD, P. A., lxx, 2.

** ROSCOE, L. E., 242; Eng. Com. L. Rep., xxv. 453; Rex *vs.* Coe, 6 CAR. & P., 403; Vaughan, 13.

bility produce the abortion, and it were proved that this fact were known to the person administering, the crime would not have been committed; on the ground that he must be presumed to have acted without the malicious intent which the law requires.* Here, however, the frequent and important physiological effect through the imagination, in producing a definite result where such is supposed to be intended, has been entirely forgotten.

In several States, a seemingly unwise discrimination has been made by the law between the various methods employed. Thus in Michigan, Mississippi, Arkansas, and Minnesota, a special statute has been enacted for cases where the crime is effected "by any injury to the mother, which would be murder if it resulted in the death of such mother." We have seen that the surest and most efficient means of producing abortion are those where no injury whatever is necessarily inflicted upon the mother. Such statutes as the above, therefore, actually tend to encourage the crime.

Another important obstacle to conviction is found in the latitude the statutes allow, generally by omission, to the plea of justification.

In Illinois and Louisiana no justification whatever is allowed by the law. In Connecticut the offence is penal where committed "willfully and maliciously;" in Vermont and Massachusetts to the latter of these expressions are added the words, "or without lawful justification:" but in each case, decision upon this point is left in great measure or wholly to the court. In Virginia, to be allowable, the abortion must have been necessary for the life of either the mother or child. In Maine, New Hampshire, Massachusetts,† New York, Ohio, Indiana, Michigan, Missouri, Alabama, Mississippi, Arkansas, Texas, Minnesota, California, Oregon, and the Territories of Kansas and Washington, it must have been performed for the sake of the mother's life alone.

In none of these States is a standard of such justification established by the statute. In Maine, New York, Indiana, Alabama, Oregon, and the Territory of Washington, this is not even attempted. In Texas, the statute acknowledges "medical advice." In Missouri, Mississippi, California, and the Territory of Kansas, the sanction of "a physician" is allowed as excuse; in Arkansas that of "a regular physician;" in New Hampshire, Ohio, Michigan, and Minnesota, "two physicians" may decide on

---

* 1 BISHOP, Crim. Law, 527.

† In Massachusetts, though the statute is silent on these points, it is asserted that whenever a potion is given, or other means are used, by "a surgeon," for the purpose of saving the life of the woman, the case is free of malice, and has a lawful justification. DAVIS, Crim. Justice, 282; Report of the Criminal Law Commissioners, 1844, Causing Abortion, I., note *a*.

its necessity; but in every one of these States, the word "or" stands engrossed upon the statute, and thereby the precaution is practically invalidated; which could not have been the case had the word "and" have been used instead.

The truth is, as we have seen, that when such latitude is allowed by the law, it is inevitably abused. Medical evidence, medical sanction, and medical performance are absolutely essential for excuse in every case; if the opinion of a single physician is allowed to be sufficient, an escape is afforded for all instances where this privilege has been dishonored; the word "regular" is much more liable to be misinterpreted than the word "competent;"* in every case before abortion can be justified, its necessity should previously have been decided and the possibility of crime thus prevented, by a consultation of at least two competent medical men.

We have pointed out many circumstances under which premature labor is demanded, by the rules of humanity and of medicine, for the sake of the mother's life, and have seen that in several States it is for this cause allowed. But we have also shown that it is often equally necessitated for the sake of the child. In no State save Virginia is its justifiability for this purpose yet recognized by the statute law.

By the codes of most States, the mother is not punishable, however directly implicated in the crime. New Hampshire, New York, and Wisconsin, are apparently the only exceptions to this statement; the last two of them also not requiring proof of pregnancy for conviction, while by implication New Hampshire does.

By the statute of Indiana, all other women equally with the mother would seem released from prosecution, the word "he" being used of the persons indictable. The same oversight is noticed in the first section of the statute of Texas.

Objections were formerly made, especially in England, to the severity of the penalties then inflicted, (death in case of quickening, transportation or long imprisonment otherwise,) on the erroneous ground that this severity was wholly disproportionate to the guilt of the offence, and that, therefore, juries did not convict. The mistaken character of this supposition is shown by abundant proof in this country. In many States the penalties of the law are absurdly insignificant, tending, equally with the uncertainty of their infliction, to encourage the crime, and yet the same difficulties of conviction prevail. The true nature of these difficulties we have endeavored in great measure to explain, and we think it will have appeared that however numerous and serious they may be, they are yet not insuperable.

* After a little reflection, it will be seen that this word is not so open to objection as might at first be supposed.

Before concluding this subject, it is proper that we examine more fully into the doctrine of the common law, to understand more precisely its meaning. In so doing, we shall quote freely from the leading authorities of the day, especially those of this country.

The destruction of an unborn child is not at the present day murder at the common law, though such was formerly the case;* to constitute which crime, the person killed must at the time of death have been alive,† as we have shown the fœtus to be from the time of conception, and "a reasonable creature in being,"‡ a quality in this connection denied to the child by the law, though in all other relations it inconsistently allows and affirms it; as it does also, and always, from the moment of birth, even though the funis is undivided and the placenta still attached.§

To cause abortion after quickening, is not, as such, murder or manslaughter at common law, but a high misdemeanor.‖

Whether to cause, or to attempt, abortion before quickening is a penal offence at common law, has been differently decided. In several of the States, as Maine, Massachusetts, and New Jersey, it has been ruled by the Supreme Court not to be indictable, even as an assault, if done with the consent of the woman; on the ground that only in case of high crimes is the person assaulted incapable of assenting.¶ The Pennsylvania court, however, has discarded this doctrine, and has decided that the moment the womb is instinct with embryo life, and gestation has begun, the crime may be perpetrated.**

The distinction alluded to with regard to quickening, is allowed by an acknowledged legal authority†† to be at open variance not only with medical experience, but with all other principles of the common law.‡‡ The civil rights of an infant in utero are respected equally throughout gestation; at every stage of which process, no matter how early, it may

---

* 1 RUSSELL, Crimes, 671; 1 VESEY, 86; 3 COKE, Inst., 50; 1 HAWKINS, C. B., s. 16; 1 HALE, 434; 1 EAST, P. C., 90; 3 CHITTY, Crim. Law, 798; WHARTON, Crim. Law, 537.

† DAVIS, Crim. Justice, 486.

‡ ARCHBOLD, Crim. Pleading, 490.

§ Regina *vs.* Trilloe, 2 MOODY, C. C., 260, 413.

‖ The State *vs.* Cooper, 2 ZABRISKIE, 52; HANES, U. S. Digest, 5.

¶ The Commonwealth *vs.* Parker, 9 METCALF, 263; The Commonwealth *vs.* Bangs, 9 Mass., 387; The State *vs.* Cooper, 2 ZABRISKIE, 57; HANES, U. S. Digest, 5; Smith *vs.* State, 33 MAINE, (3 RED.) 48.

** BISHOP, Crim. Law, 386; Mills *vs.* The Commonw., 1 HARRIS, Pa., 631, 633.

†† WHARTON, Crim. Law of the U. S., 537.

‡‡ 1 RUSSELL, Crimes, 661; 1 VESEY, 86; 3 COKE, Inst., 50; 1 HAWKINS, c. 13 s. 16; BRACTON, l. 3, c. 21.

be appointed executor,* is capable of taking as legatee† or under a marriage settlement,‡ may take specifically as "a child" under a general devise,§ and may obtain an injunction to stay waste.||

When, in an attempt to procure an abortion, there is an evident intent to produce the death of the mother, and her death does actually occur, such attempt becomes murder at common law;¶ but when nothing more is intended than to commit the misdemeanor, it is only manslaughter,** being an instance of homicide from individual malice toward a third party, when the fatal blow falls on the deceased by mistake. It has been said, however, that this last is not the true doctrine, the destruction of an infant in utero being, even at common law, in some respects felonious, and the act in its nature malicious and deliberate, and necessarily attended with danger to the person on whom it is performed.††

The use of violence upon a woman, with an attempt to produce her miscarriage without her consent, rules Chief Justice Shaw, of Massachusetts, is an assault highly aggravated by such wicked purpose, and would be indictable at common law. So where, upon a similar attempt, the death of the mother ensues, the party making such an attempt, with or without her consent, is guilty of murder, on the ground that it is an act done without lawful purpose, dangerous to life, and that the consent of the woman cannot take away the imputation of malice, any more than in case of a duel, where in like manner there is the consent of the parties.‡‡

Though to kill the fœtus in utero is as such, by the common law, no murder, yet if it be born alive, and die subsequently to birth from the wounds it received in the womb, or from the means used to expel it, the offence becomes murder in those who cause or employ them.§§ If a person, intending to procure abortion, does an act which causes the child to be born earlier than its natural time, and therefore in a state much less capable of living, and it afterwards die in consequence of such premature

---

* BAC. Ab., tit. Infants.

‡ Doe *vs.* Clark, 2 H. Bl., 399; 2 VESEY, jr., 673; Thellusson *vs.* Woodford, 4 VESEY, 340; Swift *vs.* Duffield, 6 SERG. & RAWLE, 38.

§ FEARNE, 429.

|| 2 VERNON, 710; The Commonwealth *vs.* Demain, 6 Penn. Law Journ., 29; BRIGHTLY, 441.

¶ 1 HALE, 90; The Commonw. *vs.* Chauncey, 1 ASHMEAD, 227; Smith *vs.* State, 33 MAINE, (3 RED.) 48.

** Ibid.; HANES, U. S. Digest, 5.

†† WHARTON, Law of Homicide, 44.

‡‡ The Commonw. *vs.* Parker, 9 METCALF, 263, 265; DAVIS, Crim. Justice, 281.

§§ 1 BLACKSTONE, 129; Rex *vs.* Senior, 1 MOODY, C. C., 346; 3 Inst., 50; WHARTON, C. L., 537; Ibid., Law of Homicide, 93.

exposure, the person who by this misconduct brings the child into the world, and puts it into a situation in which it cannot live, is guilty of murder, though no direct injury to the child be proved; and the mere existence of a possibility that something might have been done to prevent the death, does not lessen the crime.*

The earlier English statutes, from their peculiar phraseology, held pregnancy essential for the commission of the crime,† yet an attempt to produce abortion is now indictable at common law,‡ though it fail by reason of the woman being, in fact and contrary to the belief of the party, not pregnant.§ For though as no man would attempt what he absolutely knew he could not in fact perform, nor would be deemed in law to have so attempted, and as every one being conclusively presumed to understand the law, no man can legally intend what is legally impossible, the rule as to facts is different; for men are not conclusively held by the law to know facts. And if a man fails in what he undertakes, because of an impossibility in fact, which he did not know, he is just as answerable as if the failure were from any other cause.||

We have seen the mistaken basis, as regards the criminality of abortion, on which the common law is founded, and that while it recognizes the distinct existence of the fœtus for civil purposes, it here considers its being as totally engrossed in that of the mother.

A recent authority thus accounts for and defends the mistake. The wealth and prosperity of the country, it is assumed, and the growth and efficiency of its population, are alike matters of general concern, and therefore the law takes them under its care. As to population, there are in civil jurisprudence such rules, as that the husband may hold the lands of his deceased wife during his life, if before her death a living child was born, but not otherwise; the law thus offering, in effect, a reward for issue. It does not compel matrimony, because that would be an infringement of private rights; but for the same end, it does punish abortions.¶

Another writer has also implied that the common law, in making fœticide penal, had in view the great mischiefs which would result from even its qualified toleration: namely, the removal of the chief restraint upon illicit intercourse, and the shocks which would be sustained thereby

---

* Rex *vs.* West, 2 CARR. & KIR., 784; 1 BISHOP, C. L., 255; WHARTON, Law of Homicide, 93.

† Rex *vs.* Scudder, 1 MOODY, 216, 3 CAR. & P., 605, overruling Rex *vs.* Phillips, 3 CAMPBELL, 73; RUSSELL, Cr., 763, note.

‡ If made without her consent?

§ Regina *vs.* Goodchild, 2 CAR. & KIR., 293; Rex *vs.* Goodhall, 1 DEN. C. C., 187; 3 CAMPBELL, 76.

|| 1 BISHOP, Crim. Law, 518. ¶ Ibid., 385.

by the institution of marriage and its incidents; among which the delicacy of women.*

In unison with these opinions, Judge Coulter, of Pennsylvania, has ruled, that "it is not the murder of a living child which constitutes the offence, but the destruction of gestation."†

If our previous assumptions of the actual character of criminal abortion be granted, and we believe that they have been proved to a demonstration, it must follow from the subsequent remarks that the common law, both in theory and in practice, is insufficient to control the crime; that in many States of this Union, the statutory laws do not recognize its true nature; that they draw unwarrantable distinctions of guilt; that they are not sufficiently comprehensive, directly allowing many criminals to escape, permitting unconsummated attempts, and improperly discriminating between the measures employed; that they require proofs often unnecessary or impossible to afford; that they neglect to establish a standard of justification, and thereby sanction many clear instances of the crime; that by a system of punishments wholly incommensurate with those inflicted for all other offences whatsoever, they thus encourage instead of preventing its increase; and that in many respects they are at variance, not merely with equity and abstract justice, but with the fundamental principles of law itself.

"It is to be hoped," has forcibly been written, "that the period is not far remote, when laws so cruel in their effects, so inconsistent with the progress of knowledge and civilization, and so revolting to the feelings and claims of humanity, will be swept from our statutes."‡

In a similar trust, it now behooves us to consider whether, and in what manner, the difficulties in the way of generally suppressing the crime of abortion can be overcome.

[TO BE CONCLUDED.]

---

ART. II.—*Gangrene of the Lung, resulting from a Foreign Substance lodged in the Right Bronchial Tube, and terminating in Empyema, and Perforation through the Diaphragm and into the Colon. Final Expulsion of the Body and Recovery of the Patient.* By E. J. FOUNTAIN, M.D., of Davenport, Iowa.

THE following case presents a series of phenomena of a very rare, if not unprecedented occurrence, resulting from a foreign body in the lungs. I

---

* WHARTON, Crim. Law, 541. † 1 HARRIS, Pa., 631, 633.

‡ LEE, Note to Guy's Principles of Forensic Medicine, p. 134.

